# Prenatal glucocorticoids exposure and adverse cardiovascular effects in offspring

**DOI:** 10.3389/fendo.2024.1430334

**Published:** 2024-09-16

**Authors:** Chenxuan Zhao, Lei He, Lingjun Li, Fengying Deng, Meihua Zhang, Changhong Wang, Junlan Qiu, Qinqin Gao

**Affiliations:** ^1^ Institute for Fetology, First Affiliated Hospital of Soochow University, Suzhou, China; ^2^ Key Laboratory of Birth Regulation and Control Technology of National Health Commission of China, Shandong Provincial Maternal and Child Health Care Hospital Affiliated to Qingdao University, Jinan, China; ^3^ Genetics and Prenatal Diagnosis Center, Fuyang People’s Hospital, Fuyang, China; ^4^ Department of Oncology and Hematology, Suzhou Hospital, Affiliated Hospital of Medical School, Nanjing University, Suzhou, Jiangsu, China

**Keywords:** prenatal GCs exposure, 11β hydroxysteroid dehydrogenase 2, placental GCs barrier, cardiovascular system, offspring

## Abstract

Glucocorticoids (GCs) are steroid hormones fundamental to the body’s normal physiological functions and are pivotal in fetal growth and development. During gestation, the mother’s cortisol concentration (active GCs) escalates to accommodate the requirements of fetal organ development and maturation. A natural placental GCs barrier, primarily facilitated by 11β hydroxysteroid dehydrogenase 2, exists between the mother and fetus. This enzyme transforms biologically active cortisol into biologically inactive corticosterone, thereby mitigating fetal GCs exposure. However, during pregnancy, the mother may be vulnerable to adverse factor exposures such as stress, hypoxia, caffeine, and synthetic GCs use. In these instances, maternal serum GCs levels may surge beyond the protective capacity of the placental GCs barrier. Moreover, these adverse factors could directly compromise the placental GCs barrier, resulting in excessive fetal exposure to GCs. It is well-documented that prenatal GCs exposure can detrimentally impact the offspring’s cardiovascular system, particularly in relation to blood pressure, vascular function, and heart function. In this review, we succinctly delineate the alterations in GCs levels during pregnancy and the potential mechanisms driving these changes, and also analyze the possible causes of prenatal GCs exposure. Furthermore, we summarize the current advancements in understanding the adverse effects and mechanisms of prenatal GCs exposure on the offspring’s cardiovascular system.

## 1 Introduction

Glucocorticoids (GCs) are critical stress hormones involved in various fundamental processes, including metabolic homeostasis, cognition, and development (1, 2). They exhibit potent anti-inflammatory and immunosuppressive effects, making synthetic GCs such as dexamethasone and betamethasone, a common clinical treatment for a broad spectrum of autoimmune, inflammatory, and allergic diseases (2, 3). GCs not only serve as effective therapeutic agents for numerous diseases but also function as essential steroid hormones for maintaining the body’s normal physiological functions, particularly for fetal growth and development (4, 5). During the second and third trimester, endogenous GCs can stimulate the development and maturation of fetal organs such as the lungs and kidneys (5). This process can also be induced prematurely by exogenous GCs. Consequently, GCs are frequently administered clinically to pregnant women at risk of preterm labor to decrease the mortality rate among premature infants.

Despite the significant role of GCs in treating various diseases and maintaining physiological functions, chronic or excessive exposure to GCs has been linked to an increased risk of cardiovascular diseases (6, 7). GCs exert direct effects on the heart and blood vessels, mediated by glucocorticoid and mineralocorticoid receptors and modulated by the local metabolism of GCs via the enzyme 11β-hydroxysteroid dehydrogenase (11βHSD). This direct impact on the cardiovascular system can impair vascular and cardiac functions and potentially resulting in cardiovascular diseases (8).

Recent findings suggest that preterm infants treated prenatally with betamethasone (synthetic GCs) exhibit a transient physiological peak in circulating GCs bioactivity (9). This implies that prenatal exposure to GCs may lead to elevated fetal circulating cortisol concentrations, potentially exerting long-term effects on the cardiovascular system of the developing fetus. When there is an excessive amount of active GCs in the fetal blood circulation, it is bound to affect the development and function of the fetal cardiovascular system, and further lay the foundation for the occurrence of cardiovascular system health problems after birth.

Increasing evidence now suggests that many adult cardiovascular diseases may be linked to intrauterine growth and development (10, 11). The theory that adult cardiovascular diseases have fetal origins has been extensively corroborated by numerous studies over the past two decades (11, 12). Recently, due to the rising prevalence of prenatal GCs exposure, many researchers have sought to investigate the long-term effects of prenatal GCs exposure on the cardiovascular system of offspring (13–15). Studies have demonstrated that excessive prenatal GCs exposure can negatively impact the cardiovascular system of both the fetus and the adult offspring, particularly in relation to blood pressure, vascular function, and cardiac development (14–16). This review primarily focuses on the detrimental effects and mechanisms associated with prenatal GCs exposure on the cardiovascular system of the offspring, as well as potential contributing factors to prenatal GCs exposure and their mechanisms.

## 2 GCs synthesis and changes during pregnancy

**Figure 1:**
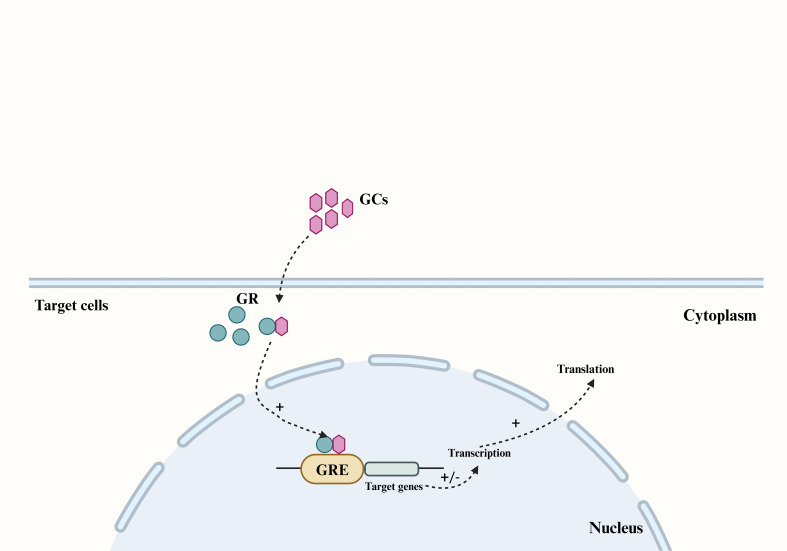
The main pathway by which GCs produce genetic effects in their target cells. The fat-soluble steroid hormones GCs can penetrate the cell membrane of target cells and bind to the GR in the cytoplasm, and cause the GR to translocate to the nucleus and bind to the GRE in the target region, thereby regulating the transcription and translation of the target gene. GCs, Glucocorticoids; GR, glucocorticoid receptor; GRE, glucocorticoid response element.

GCs are synthesized in the adrenal cortex through the enzymatic processing of cholesterol (17), with cortisol (also known as hydrocortisone) accounting for 90% of the total. GCs secretion can be categorized into basal secretion, which occurs under normal physiological conditions, and stress secretion, which occurs in response to stress. Both types of secretion are regulated by the hypothalamic-pituitary-adrenocortical (HPA) axis. In response to psychological or physiological stress, neurons in the paraventricular nucleus (PVN) of the hypothalamus synthesize corticotropin-releasing hormone (CRH) and release it into the pituitary portal vein (18). This stimulates the anterior pituitary gland to release adrenocorticotropic hormone (ACTH) into the bloodstream, which in turn triggers the synthesis and secretion of GCs from the adrenocortical zona fasciculata (19, 20). As a fat-soluble steroid hormone, GCs can easily penetrate the cell membrane and bind to the cytoplasmic glucocorticoid receptor (GR). The GR then translocates to the nucleus and binds to the glucocorticoid response element (GRE) in the target region, thereby regulating the transcription and translation of target genes and eliciting corresponding gene effects (**Figure 1**) (21, 22). Cortisol, a steroid hormone crucial for early gestational pregnancy establishment (23, 24), also plays a vital role in fetal development. A significant increase in fetal serum GCs levels before birth is essential for the normal development of fetal lungs and other organs (25). Studies have demonstrated a substantial rise in both total cortisol and biologically active plasma free cortisol concentrations in pregnant women (26, 27).

## 3 Placental GCs barrier

**Figure 2:**
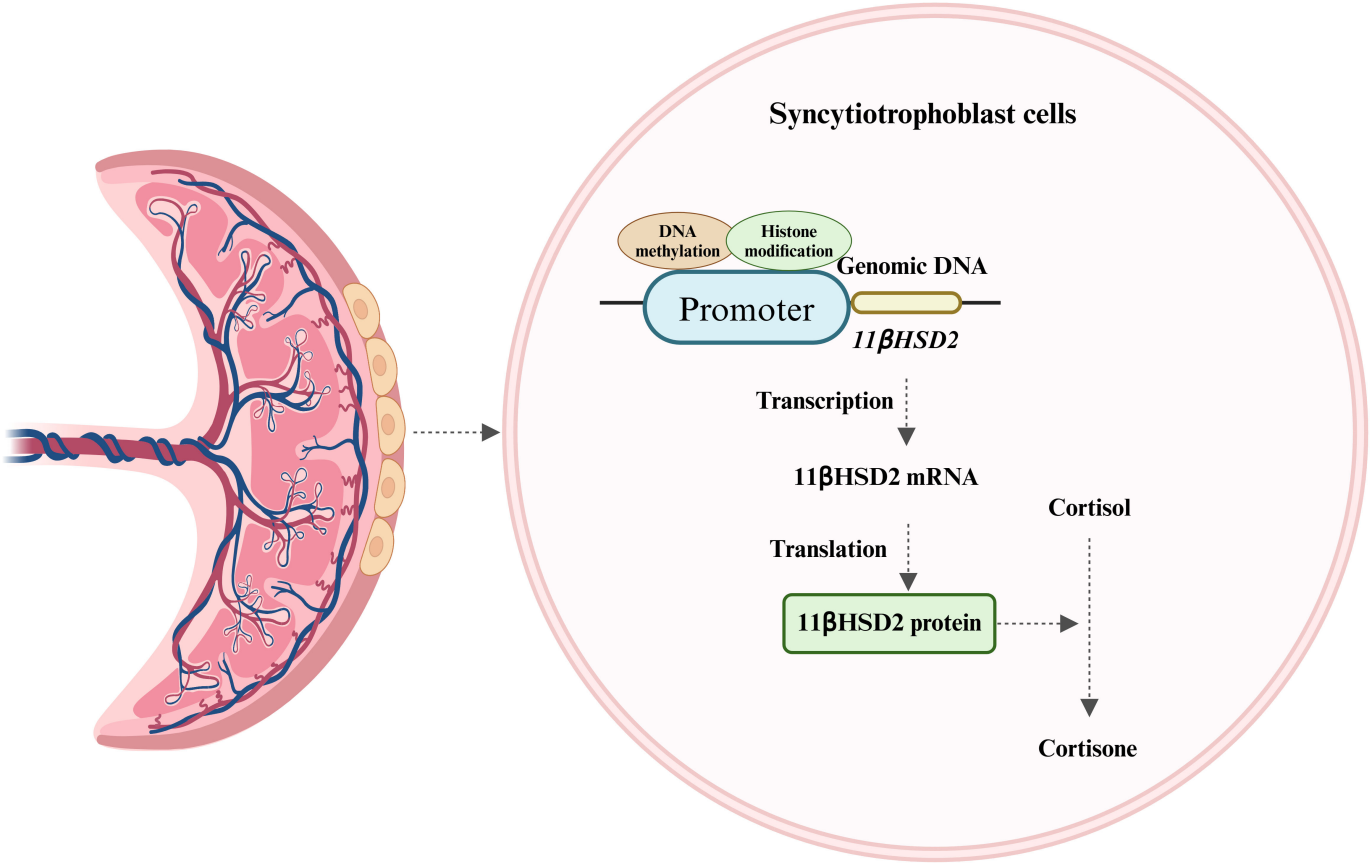
Expression of 11βHSD2 and its major role. 11βHSD2, expressed in the syncytiotrophoblast of placental villi, converts biologically active cortisol to inactive cortisone and is regulated by DNA methylation and histone modifications.

The human placenta, is characterized by the direct interface between maternal blood circulation and placental villi. These villi, the site of nutrient and gas exchange between the mother and fetus, are enveloped by two layers of trophoblasts (28). The syncytiotrophoblast, one of these layers, is situated in the intervillous space of the chorionic villi and serves as the primary defense line, shielding the fetus from potentially harmful maternal substances (28). Although the fetal HPA axis can secrete a minimal amount of cortisol in late pregnancy, approximately 50% of fetal cortisol originates from the mother via placental blood circulation. The maternal endogenous cortisol concentration during pregnancy is 5-10 times higher than that in the fetus. This concentration gradient is largely maintained by the placental GCs barrier, which is reinforced by 11β-hydroxysteroid dehydrogenase 2 (11βHSD2) (28). 11βHSD2, a glucocorticoid-inactivating enzyme, has a high affinity for cortisol and can convert biologically active cortisol into inactive cortisone, thereby protecting the fetus from the detrimental effects of maternal GCs overdose (**Figure 2**) (28). 11βHSD2 is present on the surface of the chorionic villi early in pregnancy and plays a crucial role in safeguarding embryonic development (28). As fetal age increases, the activity and expression of 11βHSD2 rise correspondingly until late gestation when it starts to decline (29, 30).

It is currently understood that the expression of 11βHSD2 is strictly regulated by several mechanisms, including DNA methylation and histone modification. The methylation pattern within the promoter of the 11βHSD2 gene is established during trophoblast fate determination (28). Rasoul, et al. reported that the methylation level of the CpG island in the promoter is a determinant of whether 11βHSD2 gene expression is repressed (31). Research has also demonstrated that the regulation of the 11βHSD2 gene is influenced by human chorionic gonadotropin (hCG) (32, 33). The specific mechanism involves hCG triggering the cAMP/PKA pathway, which in turn inactivates the PRB-e2F1-EZH2 pathway. This sequence of events results in a decrease in histone H3 lysine 27 methylation and an increase in acetylation levels at the 11βHSD2 gene promoter region. Consequently, the transcription factor specificity protein 1 (Sp1) can bind to the 11βHSD2 gene promoter, thereby enhancing its transcription (32, 33). Furthermore, studies have indicated that the activity of placental 11βHSD2 can be affected by various factors (34, 35). For instance, elevated levels of cortisol in the maternal environment during pregnancy or the prenatal use of synthetic GCs can diminish 11βHSD2 activity (9).

Given the crucial role of 11βHSD2 in the placental GCs barrier and in safeguarding the fetus from the risks of excessive GCs exposure, even minor alterations in its expression and activity can have detrimental effects on fetal development. Therefore, the mechanisms regulating the expression and activity of 11βHSD2 in the placenta warrant further investigation and clarification.

## 4 Possible causes of prenatal GCs exposure

**Figure 3:**
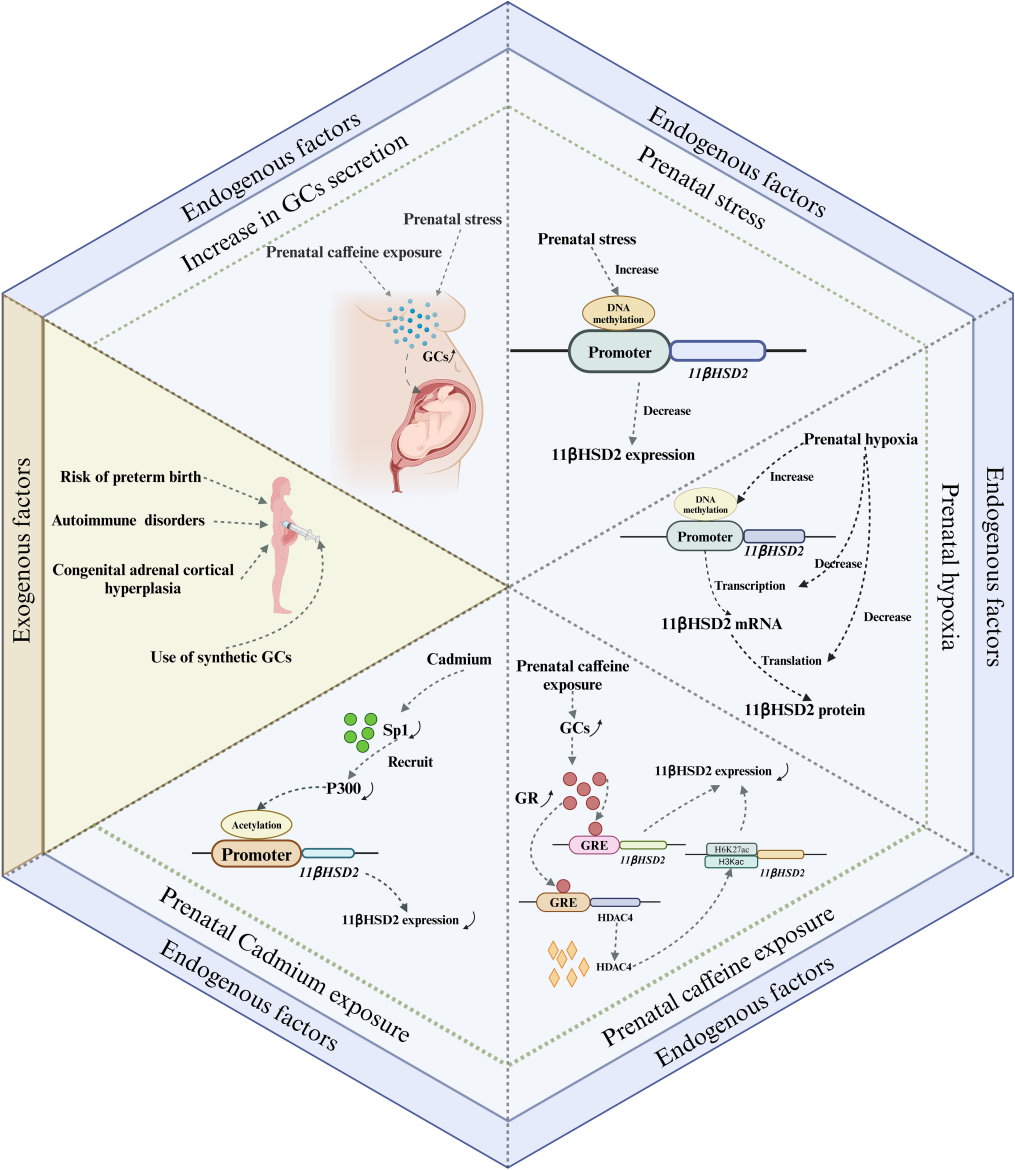
Major pathways of prenatal GCs exposure and their possible mechanisms. There are two main endogenous and exogenous routes of prenatal fetal exposure to GCs. The endogenous pathway is mainly due to overproduction of GCs and impaired placental GCs barrier, whereas the exogenous pathway is mainly due to the use of synthetic GCs, which are commonly used for the treatment of maternal immune disorders and the prevention of preterm birth complications. Among these, impairment of the placental GCs barrier caused by exposure to adverse factors occurs mainly by affecting the expression of 11βHSD2. GCs, Glucocorticoids; GR, glucocorticoid receptor; GRE, glucocorticoid response element.

During pregnancy, the placental GCs barrier provides a degree of protection to the fetus from maternal excess GCs. However, under certain exceptional circumstances, such as impaired placental barrier or maternal exposure to high levels of GCs, the fetus can be adversely affected by GCs. Prenatal GCs exposure can occur via two primary pathways: endogenous and exogenous. The endogenous pathway primarily results from overproduction of GCs and impairment of the placental GCs barrier, while the exogenous pathway involves the use of synthetic GCs, typically for maternal immune disorders and to prevent complications in preterm infants (**Figure 3**).

### 4.1 Endogenous factors

#### 4.1.1 Increase in GCs secretion

GCs are the final product of the HPA axis. Any disruption to the HPA axis can potentially lead to an abnormal surge in the body’s GCs secretion. This, in turn, may result in the fetus being exposed to excessive GCs. Maternal plasma cortisol levels are known to escalate and reach their peak during late gestation, with serum cortisol levels tripling compared to those in non-pregnant women. Concurrently, the concentrations of placental 11βHSD2 decrease in late pregnancy. This reduction allows more cortisol to permeate the placenta (36), thereby increasing the fetus’s susceptibility to excessive GCs during late gestation. Therefore, from a physiological perspective, late pregnancy presents a critical window for fetal exposure to cortisol. In addition to physiological factors, exposure to adverse prenatal conditions can also contribute to elevated maternal GCs secretion. Prenatal stress, for instance, has been shown to increase serum corticosterone concentrations of pregnant rats (37, 38). In a study by Takahashi et al., it was found that the rise in plasma corticosterone concentrations in pregnant rats, induced by prenatal stress, primarily occurred 24 and 48 hours post-stress exposure. This increase was accompanied by a significant decrease in maternal steroid-binding globulin levels, suggesting that stress primarily triggers an increase in the circulating levels of free corticosterone *in vivo* (39). In addition, Ge et al. reported that prenatal exposure to caffeine also increased maternal serum, fetal serum and placental corticosterone levels, which may be due to epigenetic regulation of the RYR/JNK/YB-1 pathway affecting cortisol efflux function (40). In conclusion, an increase in GCs secretion, driven by a combination of physiological and pathological factors, is the primary cause of fetal exposure to excessive GCs.

#### 4.1.2 Impaired placental GCs barrier

##### 4.1.2.1 Prenatal stress

Both human and animal studies indicate that prenatal acute and chronic stress may differentially impact the expression of 11βHSD2 in the placenta. Acute stress appears to stimulate placental 11βHSD2 expression (32, 41), whereas chronic stress seems to inhibit it (42–45). The up-regulation of placental 11βHSD2 expression in response to acute stress may represent an immediate protective mechanism by the fetus to counteract a sudden surge in maternal GCs. Conversely, the suppression of placental 11βHSD2 expression under chronic stress may serve as a survival strategy for the fetus, given that chronic stress often predisposes to preterm labor (46). This inhibition allows more cortisol to permeate the fetal circulation, thereby expediting the maturation of crucial fetal organs to ensure survival. However, this survival strategy is a double-edged sword, as it results in growth restriction and consequently elevates the risk of chronic disease development later in life. Currently, while the mechanism through which acute stress stimulates placental 11βHSD2 expression remains elusive, emerging evidence suggests that the down-regulation of placental 11βHSD2 expression induced by chronic stress may be associated with DNA methylation (47–49). For instance, Catherine et al. revealed that prenatal stress increased DNA methylation at specific CpG sites within the 11βHSD2 gene promoter in the placenta (48). Although the detailed mechanisms by which chronic stress during pregnancy leads to high DNA methylation of the 11βHSD2 gene are not clear, increased expression of DNA methyltransferases may be involved in this process (48).

##### 4.1.2.2 Prenatal hypoxia

Prenatal hypoxia, a common pregnancy complication, is often induced by a variety of factors including maternal, placental, and fetal conditions. This condition can disrupt the placental GCs barrier by impacting 11βHSD2 expression (50, 51). A murine study demonstrated that maternal hypoxia during mid- to late-gestation not only modified placental morphology but also resulted in reduced expressions of 11βHSD2 and GR in the placenta (50, 52). Similarly, human studies have reported a reduction in placental 11βHSD2 expression during fetal asphyxia in late-stage intrauterine growth restriction pregnancies (51). *In vitro* studies have corroborated these findings, showing inhibited induction of 11βHSD2 when cells are exposed to hypoxic conditions (53, 54). Concurrently, both maternal and fetal plasma cortisol levels, as well as the ratio of fetal to maternal plasma cortisol levels, were found to be elevated in cases of prenatal hypoxia (52). This suggests that prenatal hypoxia suppresses 11βHDS2 expression and enhances the rate of maternal cortisol crossing the placenta. Additionally, the abundance of 11βHSD2 mRNA was also diminished in placentas affected by prenatal hypoxia, indicating that the reduction in 11βHSD2 expression may be associated with transcriptional inactivation. This implies that prenatal hypoxia may reprogram the expression of the 11βHSD2 gene through DNA methylation (52).

##### 4.1.2.3 Prenatal caffeine exposure

Caffeine is widely found in coffee, tea, soft drinks, foods and medications, and it is also very common today for women to consume caffeine during pregnancy. Caffeine, due to its high fat solubility, can traverse the placental barrier and enter the fetus. Its slow metabolism and excretion within the fetus can lead to its accumulation, potentially causing detrimental effects on fetal health. One such effect is the impairment of the placental GCs barrier. This may be attributed to caffeine’s inhibitory effect on the expression of 11βHSD2 in the placenta (55), resulting in an excess of corticosterone. This, in turn, leads to elevated levels of circulating corticosterone in the fetus, thereby increasing the risk of various chronic diseases in the offspring (56–58). Currently, the exact mechanism through which prenatal caffeine exposure inhibits placental 11βHSD2 expression remains unclear. However, emerging evidence points towards the role of epigenetic modifications (59, 60).

##### 4.1.2.4 Exposure to other adverse prenatal factors

In recent years, studies have found that exposure to heavy metals during pregnancy can also damage the barrier function of the placental GCs (61, 62). For example, prenatal exposure to cadmium disrupts the placental GCs barrier, leading to fetal exposure to high levels of cortisol, ultimately affecting the health of newborns and their offspring (61, 62). Fan et al. found that prenatal cadmium exposure can affect the binding of P300 and 11βHSD2 gene promoter regions by reducing Sp1 expression, thereby reducing acetylation modifications in the 11βHSD2 gene promoter region, and subsequently inhibiting the expression of 11βHSD2 (61). Chen et al. speculated that prenatal cadmium exposure inhibits placental 11βHSD2 expression by downregulating the cAMP/PKA/Sp1 signaling pathway (62).

In addition to the above adverse factors, recent studies have found that prenatal nicotine exposure and alcohol consumption have also differentially affected the function of the placental GCs barrier to varying degrees (62, 63). For example, Chen et al. found that prenatal nicotine exposure can lead to elevated maternal serum corticosterone levels, and significantly reduced placental 11βHSD2 mRNA levels (63). Liang et al. established a prenatal alcohol exposure model in pregnant mice and found that after prenatal alcohol exposure, serum corticosterone levels significantly increased, and placental 11βHSD2 expression decreased (64). Furthermore, it is discovered that the nutritional status of the mother during pregnancy can also impact the expression of placental 11βHSD2. Generally, excessive nutrition or obesity during pregnancy results in a reduction in placental 11βHSD2 expression (65, 66), while malnutrition has no effect on the expression of 11βHSD2 in the placenta (67). Currently, although a large quantity of studies has indicated that many prenatal adverse factors lead to a decrease in the expression of placental 11βHSD2 (**Table 1**), the underlying mechanism remains unclear, and further research and clarification are urgently required in the future.

**Table 1:** Prenatal adverse factors in regulating placental 11βHSD2 expression.

Factor	Expression	Reference
Stress	Down	(42–45)
Hypoxia	Down	(49–53)
Caffeine	Down	(54, 58, 59)
Cadmium	Down	(60, 61)
Nicotine	Down	(62)
Alcohol	Down	(63)
Overnutrition	Down	(65, 66)

In contrast to the function of 11βHSD2, 11βHSD1 has the ability to convert inactive GCs into active GCs, and 11βHSD1 and 11βHSD2 jointly regulate the level of active GCs in the local tissues of the fetus. In the fetal cardiovascular system, once there is any abnormality in the expression or activity of 11βHSD1/2, it will cause a change in the level of active GCs within the cardiovascular system, and then have a long-term influence on the development of the fetal cardiovascular system and the health of the offspring’s cardiovascular system. At present, although there are relatively numerous relevant research reports on the influence of prenatal adverse factors on the expression or activity of 11βHSD1/2 in fetal tissues (68–70), the research on the fetal cardiovascular system is rather limited. Hence, this review did not carry out a detailed overview of the research progress in this aspect.

### 4.2 Exogenous factors

The main route of prenatal exogenous GCs exposure is the use of synthetic GCs. GCs play a crucial role in promoting fetal organ maturation. Particularly in pregnant women threatened with preterm labor, synthetic GCs are widely used to accelerate lung maturation, thereby greatly improving the survival of preterm neonates (64). In addition, GCs use will be unavoidable when the mother has an autoimmune disease, such as connective tissue disease. What’s more, in order to suppress the overproduction of adrenal androgens in the fetuses of pregnant women at high risk of congenital adrenocortical hyperplasia, small doses of dexamethasone are usually given to pregnant women in the early stages of gestation in clinical treatment, which is effective in reducing the male genitalia of fetuses whose mother with congenital adrenocortical hyperplasia (71, 72). In conclusion, the clinical application of antenatal GCs has inevitable characteristics, especially for premature infants. Although they can promote lung maturation in premature fetuses, increase the rate of live births, and reduce the incidence of neonatal respiratory distress syndrome, their use also has long-term adverse effects on maternal and infant health.

## 5 Effects of prenatal GCs on offspring cardiac functions

Currently, a considerable amount of research indicates that prenatal GCs exposure can have long-term adverse effects on the heart development and function of fetuses and offspring (72, 73). For example, Langdown et al. found that offspring exposed to prenatal dexamethasone exhibit disrupted cardiac glucose metabolism, along with significant activation of the Akt/protein kinase B pathway and upregulation of GLUT1 expression in cardiac tissues (74). GLUT1 is a glucose transporter and a major mediator of basal glucose uptake in the heart. They further demonstrated that prenatal dexamethasone exposure upregulates cardiac GLUT1 expression through activation of the Akt/PKB pathway, leading to disrupted cardiac glucose metabolism in offspring (74). Cardiac uncoupling protein (UCP)-mediated metabolic adaptations define cardiac cell function in the heart (75), they found that prenatal GCs exposure also affects the expression of UCP in the hearts of fetal and adult offspring. Male fetuses exposed to prenatal GCs showed significantly increased expression of cardiac UCP2 and UCP3 proteins after birth, however, in adult male offspring exposed to GCs prenatally, the expression of UCP2 and UCP3 proteins was significantly decreased (76). In addition, overexpression of the conserved Ca^2+^-binding protein calreticulin impairs cardiac function (77). Normally, cardiac calreticulin expression decreases between 2-3 weeks of age and remains suppressed into adulthood. Langdown et al. observed that the impact of prenatal dexamethasone exposure on postnatal cardiac calsequestrin expression is minimal, but the decrease in postnatal cardiac calreticulin expression is eliminated, and adult cardiac calreticulin expression significantly increases (78). Given the known association between excessive cardiac calreticulin expression and impaired cardiac function, upregulation of cardiac calreticulin may increase the risk of adult heart disease due to prenatal overexposure to GCs.

Prenatal GCs exposure was also reported to alter the development of cardiac adrenergic and sympathetic processes in offspring (79). Bian et al. found that rat offspring exposed to prenatal dexamethasone had insufficient and decreased levels of norepinephrine, indicating that prenatal GC exposure can impair the development and activity of the fetal heart sympathetic nerve projections (79). Furthermore, their study also found that prenatal exposure to high doses of GCs interferes with the development of β-adrenergic receptor-mediated cell signaling in the rat offspring heart, enhances dose-dependent stimulation of adenylyl cyclase activity mediated by β-receptors, and increases cardiac adenylyl cyclase response (80). Similar to the effects observed in rodents, Vries et al. found that prenatal dexamethasone exposure can lead to changes in cardiac metabolism and HPA axis function in chlorocebus aethiops offspring (81). In order to further reveal the repair capacity of offspring hearts exposed to prenatal GCs for “secondary hits” such as ischemia-reperfusion (I/R), researchers constructed an I/R model (82, 83). Peng et al. found that compared to the normal group, offspring exposed to GCs prenatally after I/R had more pronounced cardiac dysfunction due to increased apoptosis of myocardial cells. They further found that bone morphogenetic protein 4 (BMP4), which is involved in myocardial cell differentiation and development pathways, was significantly downregulated in myocardial cells of offspring exposed to prenatal GCs, with a significant increase in DNA methylation in the gene promoter region (82). Prenatal GCs exposure inhibited BMP4 expression in offspring myocardial cells, thereby inhibiting the binding activity of the transcription factor HIF-1α induced by myocardial ischemia, weakening the protective effect of BMP4 on myocardial cells, ultimately resulting in more pronounced cardiac dysfunction after I/R (83). These results suggest that excessive prenatal GC exposure increases the susceptibility of offspring hearts to “secondary hits” such as I/R, due to impaired the protective effect of BMP4 on myocardial cells caused by high methylation of the BMP4 promoter region. The application of DNA methylation inhibitors may be a potential therapeutic approach for the cardiac dysfunction in offspring exposed to prenatal GCs (82, 83).

**Figure 4:**
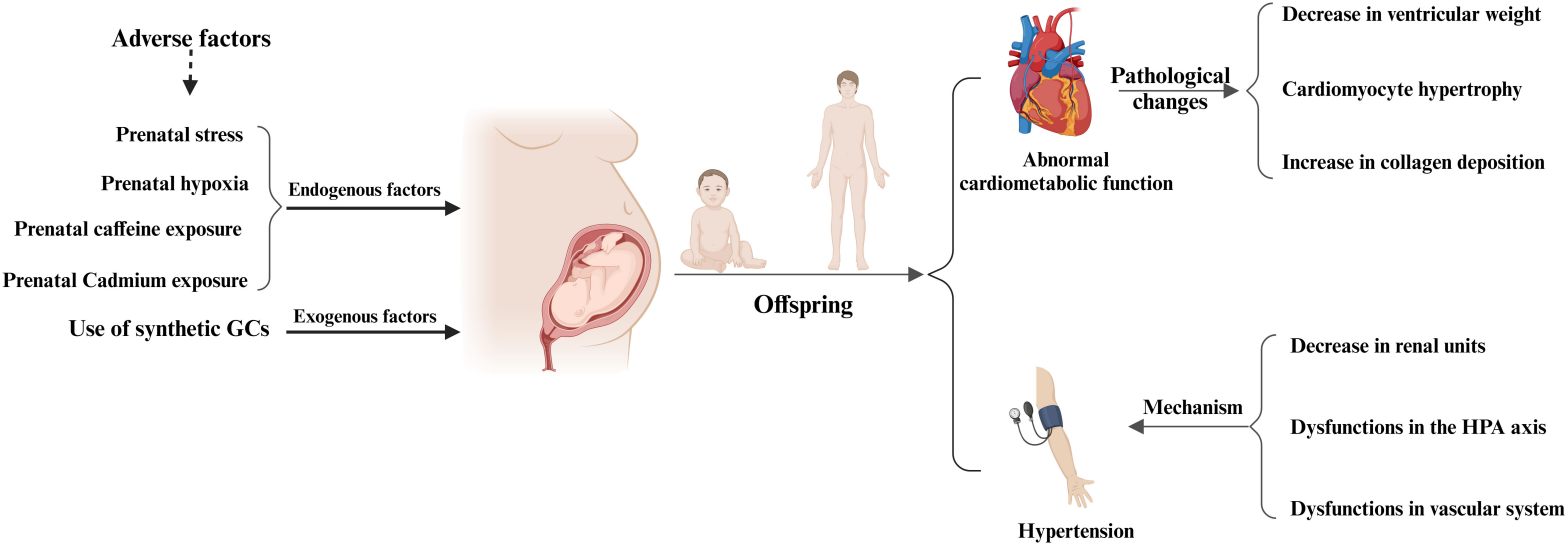
Prenatal glucocorticoids exposure and adverse cardiovascular effects in offspring. Maternal exposure to adverse factors or the use of synthetic GCs during pregnancy can adversely affect the cardiovascular system of the offspring, mainly in the form of abnormal cardiac function in the offspring, pathologic changes in the adult heart, and the development of hypertension in the offspring. Pathologic changes in the adult heart were mainly characterized by a significant decrease in ventricular weight, cardiomyocyte hypertrophy, and increased collagen deposition, whereas the development of hypertension in the offspring was mainly associated with a decrease in renal units, abnormal HPA function, and vascular dysfunction.

In conclusion, prenatal GCs exposure not only has adverse effects on the myocardium and cardiac function of newborns and offspring, but also leads to pathological changes in myocardial tissue in newborns and offspring (**Figure 4**, **Table 2**). For example, offspring exposed to prenatal GCs show a significant decrease in ventricular weight, myocardial cell hypertrophy, and increased collagen deposition (84). Although there is a considerable amount of research in this area currently, it mainly focuses on the description of pathological phenomena. Further research is urgently needed to uncover and elucidate the underlying molecular mechanisms.

**Table 2:** Effects of prenatal GC exposure on the offspring’s heart and possible genes involved.

Model	Gene	Outcome	Reference
Rat	GLUT1 (up)	Abnormal cardiac glucose metabolism	(74)
Rat	UCP (down)	Abnormal function of heart cells	(76)
Rat	BMP4 (down)	Increased susceptibility of offspring hearts to “second strikes” such as I/R	(83)
Mouse	BMP4 (down)	Increased susceptibility of offspring hearts to “second strikes” such as I/R	(82)

## 6 Effects of prenatal GCs exposure on the offspring vascular system

Currently, a large number of clinical investigations and animal experiments have studied the adverse effects of prenatal GCs exposure on the vascular system of fetal and offspring. In this chapter, we will focus on summarizing the latest research progress on the changes in blood pressure and mechanisms of prenatal GC exposure in fetuses and offspring (**Figure 4**).

### 6.1 Clinical cohort follow-up evidences

In clinical practice, GCs are often used prenatally to prevent complications in preterm infants. A large number of clinical follow-up studies have investigated the long-term effects of prenatal GCs therapy on the vascular system of infants, children, and adults (85–87). Mildenhall et al. found that in infants exposed to more than one course of prenatal corticosteroids, 67% had blood pressure above the normal range, while in infants exposed to a single course of corticosteroids, 24% had blood pressure above the normal range (85). Huh et al. tracked nearly 300 premature infants and found a positive correlation between GCs levels in umbilical cord blood at birth and systolic blood pressure at age 3 (86). However, McKinlay et al. found in a follow-up study of 320 premature children that repeated doses of betamethasone exposure prenatally did not increase blood pressure in school-age children compared to a single course of betamethasone exposure (87). Doyle et al. conducted a cohort study of 210 preterm infants and evaluated the relationship between prenatal corticosteroid therapy in preterm infants and blood pressure at age 14. Children exposed to prenatal corticosteroids had higher systolic and diastolic blood pressure compared to children not exposed to corticosteroids (88). This suggests that prenatal corticosteroid therapy is associated with increased systolic and diastolic blood pressure in adolescence and may lead to clinical hypertension after birth. Dessens et al. conducted a randomized controlled trial on subjects receiving GCs and placebo treatment, recording blood pressure in 81 subjects aged 20 (89). They found that subjects who received one course of prenatal corticosteroid therapy had lower average systolic blood pressure (89). Dalziel et al. conducted a 30-year follow-up study and found that offspring exposed to betamethasone prenatally did not have significant differences in blood pressure and cardiovascular disease history compared to the placebo group (90).

In conclusion, current clinical follow-up experimental results indicate that premature infants who have undergone prenatal GCs therapy have significantly higher blood pressure during infancy, childhood, and adolescence compared to the normal age-matched blood pressure range. However, current clinical follow-up cohorts have not found a correlation between prenatal GCs therapy and adult blood pressure. Nevertheless, due to the long follow-up time, small number of participants, heterogeneity, and varying follow-up quality, further research with larger sample sizes and higher quality follow-up cohorts is needed to investigate the relationship between prenatal GCs therapy and adult blood pressure.

### 6.2 Animal model experimental evidences

The time required for animal studies is much shorter and easier to explore the mechanisms behind diseases compared to clinical studies. Therefore, many animal models of prenatal GCs exposure have been developed, primarily in sheep and rodents. Recent experimental findings suggest that prenatal GCs exposure may result in elevated blood pressure in offspring (91, 92). In a sheep model of prenatal GCs exposure, dexamethasone (0.28 mg/day/kg) was administered from days 22 to 29 of gestation. The sheep offspring exhibited significantly elevated blood pressure from 4 to 19 months of age (93).Another study demonstrated that even a brief exposure to relatively higher levels of cortisol within the normal physiological range in the early stage of gestation (where plasma cortisol concentrations rose from 51 μmol/L to 390 μmol/L) led to increased basal mean arterial pressures in the offspring of sheep, independently of the sex of the offspring (94). Similarly, guinea pig models of prenatal GC exposure showed that prenatal dexamethasone exposure increased mean arterial pressure in mature male guinea pig offspring (16). In the Sprague-Dawley rat model, prenatal dexamethasone exposure also resulted in hypertension in offspring rats at 16 weeks of age (95). Apart from sheep and rodents, prenatal GCs exposure has been found to elevate blood pressure in non-human primates such as baboons (92, 96) and long-tailed black-jawed monkeys (81).

Overall, animal experiments have generally confirmed that prenatal GCs exposure leads to elevated blood pressure in offspring, with a potential sex difference in the susceptibility to GCs exposure: male offspring appear to be more vulnerable compared to females (97). For example, Alhamoud et al. found that adult male offspring of rats treated with prenatal dexamethasone had significantly higher blood pressure compared to the control group, while this effect was not observed in female offspring. Furthermore, protein or albumin excretion was also higher in the male offspring of the prenatal dexamethasone-treated group. These results suggest a sex difference in the impact of prenatal dexamethasone exposure on offspring blood pressure, with male offspring being more susceptible (97). Similarly, Khurana et al. conducted a study using a rat model and confirmed the presence of a sex difference in the effects of prenatal GCs exposure on offspring blood pressure. Male offspring exposed to prenatal GCs showed a more significant increase in blood pressure compared to female offspring (98).

Interestingly, the timing of prenatal GCs exposure also played a role in the development of hypertension in offspring. Exposure to dexamethasone during days 26 to 28 of gestation led to elevated basal mean arterial pressure in sheep offspring (93, 99), while exposure late in gestation did not have a significant effect (100). In addition, the effect of prenatal GCs exposure on offspring blood pressure appears to be related to the dose of GCs exposure. Prenatal exposure to a low dose of dexamethasone (10 μg/kg/d) did not significantly impact blood pressure in female mice offspring (98). These findings suggest that there is a threshold for the effects of GCs on offspring blood pressure, and using low doses may have a lesser impact on the offspring. Therefore, when using synthetic GCs for prenatal treatment, it is important to consider both the amount and duration of their use.

### 6.3 Mechanism of elevated blood pressure in prenatal GCs-exposed offspring

It is known that the occurrence of hypertension is closely related to impaired kidney development, impaired vascular structure and function, as well as abnormalities in the HPA axis. In this chapter, we will focus on summarizing the molecular mechanisms by which prenatal GCs exposure leads to elevated blood pressure in offspring (**Table 3**).

**Table 3:** Possible mechanisms and associated genes for elevated blood pressure in offspring due to prenatal GC exposure.

Model	Mechanism	Gene	Reference
Rat	Abnormal adrenaline secretion	PNMT (up)	(98, 114, 115)
Rat	Impairment of adrenal circadian signaling	/	(116)
Rat	Decrease in the number of renal units	/	(103)
Rat	Decrease in the number of renal units	AT1R and AT2R (up)	(106)
Human	Decreased synthesis of NO	/	(122)
Sheep	Increased vascular sensitivity to ET1	/	(119)
Sheep	Decrease in the number of renal units	/	(100, 105)
Sheep	Renal unit deficiency and increased expression of renal sodium channels	/	(104)
Sheep	Increased sensitivity of coronary arteries to Ang II-induced contraction	AT1R (up)	(125)
Rat	Vasodilator dysfunction	BK subunits α and β1 (down)	(15)
Rat	Increased vascular sensitivity to vasoconstrictors	Cav1.2 (up)	(126)

#### 6.3.1 Decreases in renal units

There is mounting evidence that a reduced number of renal units at birth significantly contributes to adult susceptibility to hypertension (101, 102). Ortiz et al. observed that the peak postpartum blood pressure in rat offspring, due to prenatal GCs exposure, corresponded with the period of maximum reduction in renal units (103). A study on prenatal dexamethasone-exposed sheep offspring revealed that prenatal exposure to dexamethasone led to a deficiency of renal units in the offspring, accompanied by an increased expression of fetal renal sodium channels, which persisted in the offspring (104). Furthermore, Wintour et al. discovered that the reduction in renal units in prenatal GCs-exposed offspring was linked with grossly enlarged and dilated proximal tubules, as well as an increased accumulation of collagen type I and III in the tubular interstitium and periadventitia of the renal cortical vessels (105). These studies collectively suggest that prenatal GCs exposure can disrupt fetal kidney development, leading to a decrease in the number of nephrons in offspring after birth. This, in turn, results in elevated blood pressure in the offspring. In addition, the inhibition of the Renin-Angiotensin System (RAS) during kidney development is thought to be the primary cause of the decrease in nephron number (100, 106). Moritz et al. reported that exposure to high concentrations of dexamethasone in fetal lambs can lead to significant changes in the fetal kidney RAS (100). Singh et al. reported that prenatal exposure to corticosterone in rat embryos can amplify the expression of angiotensin receptors, potentially limiting the growth and proliferation of kidney cells in offspring, thereby leading to a reduction in nephron units (106). Shalout et al. found that candesartan could normalize blood pressure in offspring exposed to prenatal betamethasone by inhibiting the expression and function of angiotensin receptors (107). Taken together, the coarsening and dilatation of the proximal renal tubules, the increased accumulation of type I and type III collagen in the tubulointerstitial and perivascular endothelium of the renal cortex, and the suppression of the RAS are all potential mechanisms through which exposure to prenatal GCs results in reduced renal units in the offspring and accordingly leads to hypertension in the offspring.

#### 6.3.2 Dysfunctions in the HPA axis

The HPA axis plays a crucial role in regulating the circulating levels of GCs and is the primary neuroendocrine system in mammals that provides a rapid response and defense against stress. Dysfunction of the HPA axis is considered an important factor in the development of hypertension. Clinical and animal studies in recent years have shown that prenatal GCs exposure not only leads to dysfunction of the fetal HPA axis, but also has long-term adverse effects on the HPA axis function of offspring after birth (108–111). For example, Matthews et al. found that prenatal GCs exposure can permanently alter the HPA function and other endocrine system regulations in offspring before puberty, during puberty, and in aging in a gender-dependent manner (112). HPA axis dysfunction is typically manifested as abnormal secretion of epinephrine. Excessive secretion of epinephrine is closely related to the occurrence of hypertension. Several studies have shown that offspring exposed to prenatal GCs have abnormal secretion of epinephrine and related gene expression, which may be important reasons for the development of hypertension in offspring exposed to prenatal GCs (98, 113, 114).

Phenylethanolamine N-methyltransferase (PNMT) is an enzyme involved in the biosynthesis of epinephrine. Nguyen et al. reported that male rat offspring exposed to prenatal dexamethasone had elevated blood pressure, and increased plasma epinephrine levels and PNMT expression (98, 114). Lamothe et al. found that DNA methyltransferases (DNMTs) and histone deacetylases (HDACs) are involved in regulating PNMT expression. To further elucidate the direct relationship between PNMT expression, and the development of hypertension in prenatal GCs-exposed offspring, they evaluated the effects of the HDAC and DNMT inhibitors on blood pressure and PNMT expression. They found that both inhibitors successfully reduced PNMT expression and epinephrine levels, and restored blood pressure to normal levels in prenatal GCs-exposed offspring (115). These results further suggest that increased PNMT gene expression and dysfunction of the HPA axis are important reasons for the development of hypertension in prenatal GCs-exposed offspring. In addition, Tharmalingam et al. performed a whole transcriptome analysis of adrenal gland of prenatal GCs-exposed offspring. They found that prenatal GCs-exposed adrenal glands have impaired circadian signaling, and suggested that changes in adrenal circadian rhythms may be a potential molecular mechanism contributing to the development of hypertension (116).

#### 6.3.3 Dysfunctions in vascular system

Blood pressure is influenced by multiple factors, and besides being related to kidney and the HPA axis, the regulation of blood pressure cannot be separated from vascular functions. Hypertension is closely related to dysfunction in vascular constriction and dilation (117, 118). It can be inferred that the increase in blood pressure in offspring caused by prenatal GCs exposure may be related to vascular dysfunction. Endothelial cells are single-layer flat cells covering the inner surface of blood vessels, capable of secreting various factors involved in the regulation of vascular function and blood pressure. Endothelin-1 (ET1) is the most effective vasoconstrictor secreted by endothelial cells. Lee et al. found that ET1 caused stronger vasoconstriction in sheep arteries exposed to prenatal dexamethasone (119). Nitric oxide (NO) is the most significant vasodilator secreted by endothelial cells, and its abnormal synthesis is related to the progression of hypertension (120, 121). Evidence suggests that one of the mechanisms by which GCs lead to vascular dysfunction is that excessive GCs can promote oxidative stress in vascular tissue, which can disrupt the availability of endothelial NO by reducing the synthesis of NO and promoting its degradation, leading to vascular dysfunction in patients with GCs excess (122). Molnar et al. evaluated the vascular endothelial function of prenatal GCs-exposed offspring and found that NO synthesis capacity was significantly reduced, and NO-dependent vascular dilation function was decreased (123). Although the mechanism is not yet clear, given the importance of NO and other endothelial secretory factors in blood pressure regulation, this provides important information for understanding the mechanisms by which prenatal GCs exposure leads to hypertension in offspring.

Vascular smooth muscle cells are the main cell type in blood vessels, and their sensitivity to classical vasoconstrictors determines the vascular resistance and regulates blood pressure. As the main effector of the RAS, Angiotensin II (Ang II) is a classic vasoconstrictor, which mainly causes vasoconstriction and blood pressure elevation through its receptor AT1R. Candesartan is an AT1R blocker which prevents Ang II from binding to them, thereby reducing blood vessel constriction and lowering blood pressure (124). Roghair et al. found that prenatal GCs exposure upregulates the expression of coronary artery AT1R receptors, enhancing the sensitivity of coronary arteries to Ang II-induced constriction (125).

BK (high-conductance Ca^2+^-activated K^+^ channels) and Cav1.2 (L-type Ca^2+^) channels are the main K^+^ and Ca^2+^ ion channels, and play an important role in vascular constriction and dilation function. Xu et al. found that prenatal GCs exposure reduces the expression of BK subunits α and β1, causing dysfunction in BK channel-mediated vascular dilation in fetal and adult offspring (15). In addition, Xu et al. also found that prenatal GCs exposure increases Cav1.2 expression, further increasing the sensitivity of blood vessels to classical vasoconstrictors such as prostaglandins and serotonin in fetal and adult offspring (126). Molecular mechanism studies have revealed that the abnormal expression of BK and Cav1.2 in offspring vascular system caused by prenatal GCs exposure is related to DNA methylation and histone modification (15, 126, 127). The above studies suggest that prenatal GCs exposure reprograms the expressions of key vascular regulatory factors in the fetal vascular system through epigenetic modifications, which accompany the offspring throughout their lives and affect the vascular functions, leading to an increase in blood pressure in offspring.

## 7 Conclusions

GCs, products of the HPA axis, play a crucial role in promoting fetal growth, development, and organ maturation when present in appropriate amounts. In almost all placental mammals, placental GCs barrier is enforced by 11βHSD2, which primarily functions to shield the fetus from an excess of maternal GCs. However, when maternal circulating GCs levels are excessive or placental GCs barrier is compromised, the fetus may be exposed to an excess of GCs. Prenatal GCs exposure can occur through two primary pathways: endogenous and exogenous. The endogenous pathway is primarily a result of GCs overproduction and impairment of the placental GCs barrier. The exogenous pathway typically involves the administration of synthetic GCs, often used to manage maternal immune disorders and prevent complications in preterm infants. There is substantial evidence suggesting that prenatal exposure to adverse factors such as stress, hypoxia, caffeine, and nicotine can impair the placental GCs barrier via reducing 11βHSD2 expression, leading to intrauterine fetal GCs exposure. Consequently, prenatal GCs exposure is characterized by its diversity, concealment, ubiquity, and at times, unavoidable clinical application.

Currently, a large number of clinical studies and animal experiments have confirmed that prenatal GCs-exposed offspring is often manifested as abnormal myocardial function and elevated blood pressure (15, 90, 126). Most of the current research on the cardiac development and function is descriptive of pathological phenomena, and further research is urgently needed to reveal and elucidate its potential mechanisms. Compared to the heart, research on the blood pressure is more comprehensive. Based on the results of clinical and animal studies, it is known that prenatal GCs exposure affects the blood pressure of newborn offspring to a certain extent (128, 129). However, due to the relative lack of clinical research data, the impact of prenatal GCs exposure on the blood pressure of human adult offspring is not yet clear. In sheep and rodent animal models, current results confirm that prenatal GCs exposure leads to elevated blood pressure in adult offspring, with a certain gender difference, as male offspring are more susceptible to the effects of GCs exposure. Additionally, the timing and dosage of prenatal GCs exposure also affect the blood pressure response of adult offspring. Further research indicates that the effects of prenatal GCs exposure on offspring blood pressure are related to various mechanisms such as reduced renal unit, HPA axis and vascular dysfunction.

Through reviewing and summarizing the related research, it can be confirmed that prenatal GCs exposure is closely related to the occurrence of cardiovascular diseases in offspring. The current findings mainly come from animal experiments. Because there are certain differences between animals and humans in terms of physiology and pathology, the experimental results cannot be completely equivalent to the human situation. Therefore, there is an urgent need to further prove it through a larger sample size and higher-quality clinical cohort follow-up investigations. Increasing evidence suggests that environmental stress can cause a series of trait changes in the parental generation (F0), some of which can be passed on to the offspring (F1) and even to subsequent generations (F2 or more). GCs are important stress hormones secreted by the body in response to external stress stimuli. Whether the pathological traits of the cardiovascular system in F1 offspring caused by prenatal GCs exposure can be transmitted across generations and the related mechanisms are still blank and worth exploring and revealing. Furthermore, the specific adverse effects and mechanisms in the fetal and offspring cardiovascular system caused by prenatal GCs exposure still need further exploration and clarification. Obtaining more information from mechanistic studies will not only understand the early developmental origins of cardiovascular diseases, providing new theoretical knowledge for the early prevention and treatment of such diseases, but also provide new insights for clinical intervention to mitigate the negative effects of prenatal GCs application. At the same time, it will also help establish new prenatal GCs treatment regimens, including optimal formulations, administration timing, and efficacy at different stages of pregnancy.
